# Early Viability

**Published:** 1851-10

**Authors:** 


*Early Viability.*—A lady, aged thirty-five, was confined, two months and seventeen days after having been driven over a rough road in a badly hung vehicle, of a very weak and small child; the whole extent of the gestation, calculated under circumstances very favorable to accuracy, had been exactly six months and ten days. The skin of the child was hardly formed of consolidation; there was no hair; the toes looked like a row of small pearls at the extremity of each foot, the fingers were so small that they were compared to lucifer matches; in fact, Dr. Ducos hardly thought the child could live.

As it seemed, however, inclined to swallow, toast-and-water mixed with milk was given. For the first ten days only, two tablespoonfuls of this fluid were given daily, the child always kept close to the fire, When two day old the infant took the breast, but it stopped at every second act of suction, and these were so feeble that the mother hardly perceived them. She was therefore obliged to procure another child to empty the breasts. In this weak state the little creature continued until the time which would have been the natural term of gestation, without increasing much in size, but a little in weight. Six weeks after this time it began now and then to smile, being then full four months old, and has since been thriving satisfactorily.—*London Lancet, from Gazette des Hôpitaux.*

